# DES-Amyloidoses “Amyloidoses through the looking-glass”: A knowledgebase developed for exploring and linking information related to human amyloid-related diseases

**DOI:** 10.1371/journal.pone.0271737

**Published:** 2022-07-25

**Authors:** Vladan P. Bajic, Adil Salhi, Katja Lakota, Aleksandar Radovanovic, Rozaimi Razali, Lada Zivkovic, Biljana Spremo-Potparevic, Mahmut Uludag, Faroug Tifratene, Olaa Motwalli, Benoit Marchand, Vladimir B. Bajic, Takashi Gojobori, Esma R. Isenovic, Magbubah Essack

**Affiliations:** 1 Institute of Nuclear Sciences “VINCA", Laboratory for Radiobiology and Molecular Genetics, University of Belgrade, Belgrade, Republic of Serbia; 2 Computational Bioscience Research Center (CBRC), King Abdullah University of Science and Technology (KAUST), Thuwal, Kingdom of Saudi Arabia; 3 Department of Physiology, Faculty of Pharmacy, University of Belgrade, Belgrade, Serbia; 4 Department of Pathobiology, Faculty of Pharmacy, University of Belgrade, Belgrade, Serbia; 5 Saudi Electronic University (SEU), College of Computing and Informatics, Madinah, Kingdom of Saudi Arabia; 6 New York University, Abu Dhabi, UAE; 7 Biological and Environmental Sciences and Engineering Division (BESE), King Abdullah University of Science and Technology (KAUST), Thuwal, Kingdom of Saudi Arabia; Marche Polytechnic University, ITALY

## Abstract

More than 30 types of amyloids are linked to close to 50 diseases in humans, the most prominent being Alzheimer’s disease (AD). AD is brain-related local amyloidosis, while another amyloidosis, such as AA amyloidosis, tends to be more systemic. Therefore, we need to know more about the biological entities’ influencing these amyloidosis processes. However, there is currently no support system developed specifically to handle this extraordinarily complex and demanding task. To acquire a systematic view of amyloidosis and how this may be relevant to the brain and other organs, we needed a means to explore "amyloid network systems" that may underly processes that leads to an amyloid-related disease. In this regard, we developed the DES-Amyloidoses knowledgebase (KB) to obtain fast and relevant information regarding the biological network related to amyloid proteins/peptides and amyloid-related diseases. This KB contains information obtained through text and data mining of available scientific literature and other public repositories. The information compiled into the DES-Amyloidoses system based on 19 topic-specific dictionaries resulted in 796,409 associations between terms from these dictionaries. Users can explore this information through various options, including enriched concepts, enriched pairs, and semantic similarity. We show the usefulness of the KB using an example focused on inflammasome-amyloid associations. To our knowledge, this is the only KB dedicated to human amyloid-related diseases derived primarily through literature text mining and complemented by data mining that provides a novel way of exploring information relevant to amyloidoses.

## 1. Introduction

Amyloid refers to aberrant extracellular proteins that clump together, forming fibrils [1, 2]. The formation of these fibrillar assemblies causes the native secondary structure of proteins to change its shape to predominantly cross-β-sheet secondary structures essential for fiber formation [3–5]. Unlike normal fibrous proteins that provide structural support in cells, these amyloid types (protein aggregates) are associated with the pathology of almost 50 disorders with different symptoms collectively referred to as amyloidoses [6–10]. Rambaran *et al*. [11] and Sipe *et al*. [12] provide inventories of amyloids associated with these human diseases, and these works show that the number of known amyloids increased from 20 in 2008 to 31 in 2016. Amyloids display diversity in structure [13], aggregation time, and site of action [14]. Thus, today’s amyloidosis represents all diseases with misfolded aggregated proteins or peptides as the common denominator.

Amyloidoses have been sub-classified into prion diseases and non-prion diseases; prions (misfolded proteins) become a self-perpetuating infectious agent in prion disease, i.e., they are transmissible, whereas misfolded proteins in non-prion diseases are non-transmissible [15]. Thus far, all amyloids do not have demonstrated infectivity as prions. However, a preformed fibril can seed amyloid formation. That is, in cell culture, seeding aggregation has been demonstrated for non-prion disease amyloids such as Tau [16–18], α-synuclein [19–21], amyloid-beta (Aβ) [22–24], huntingtin [25, 26], superoxide dismutase 1 (SOD1) [27, 28] or TDP-43 [29, 30]. Moreover, Tau [16, 20, 31–34], α-synuclein [19, 35, 36], and Aβ [23, 37] further exhibit trans-cellular propagation and the ability to induce progressive pathology *in vivo*. Our understanding of these amyloids’ roles is further complicated by experimental results showing that amyloids can interact to aggregate into hybrid amyloid fibrils (a process called cross-seeding) [38]. Reports of such co-localized amyloids include 1/ Aβ and human islet amyloid polypeptide (hIAPP) in the brain and beta-pancreatic cells [39, 40], 2/ Aβ and SAA in AD plaques [41], and 3/ Aβ and phosphorylated tau (p-tau) in synaptic terminals of AD brains [42].

To expand our insights into amyloids mechanisms of action and roles, several methods/tools have been developed to predict the propensity of proteins to aggregate, examples being Tango [43], ZipperDB [44, 45], Pasta [46], NetCSSP [47], FoldAmyloid [48], AmyloidMutant [49, 50], AmylPred2 [51]. When developing such tools/methods, the key task is to find approaches for discovering sequence segments responsible for self-aggregation and protein destabilization [44, 52–54]. Other databases facilitate in-depth investigation of these amyloids due to their link to several disease states. Representative databases include AMYPdb, (Amyloid Protein Database) [55], CPAD (Curated Protein Aggregation Database) [56], and ALBase (Amyloid Light Chain Database) [57] [http://albase.bumc.bu.edu/aldb/]. Specifically, the AmyPDB database houses amyloid protein data from 33 amyloid families, including 1,705 proteins, complemented with bibliographic references and 3D structures. While CPAD provides a collection of more than 2300 experimentally observed aggregation rates for known amyloids that can be accessed based on various classifications. This data is further linked to Uniprot [58], Protein Data Bank [59], PubMed [60], GAP [61], TANGO [62] and WALTZ [63]. On the other hand, ALBase contains 4364 amyloid nucleotide sequences, of which 808 encode monoclonal proteins that form fibrillar deposits in patients with light chain, amyloidosis, including 295 control light chain sequences from healthy subjects used to analyze the amyloid sequences. Overall, the existing amyloid-related databases’ scope is restrictive and does not allow for the comprehensive exploration of literature information and all biomedical terms/concepts related to amyloids.

Here we present DES-Amyloidoses (https://www.cbrc.kaust.edu.sa/des-amyloidosis/), a text and data mining-based KB developed to facilitate more efficient exploration of information contained in the literature and link concepts related to human amyloidosis. The development strategy is similar to the one we presented in [70]. Specifically, for text indexing, we used pre-compiled biomedical terms/phrases (referred to as concepts) organized into thematic dictionaries (e.g., diseases, pathways, miRNAs, lncRNAs, and so forth). The KB searches for the dictionary terms in titles, abstracts, and full-length articles retrieved from PubMed and PubMed Central [37]. We incorporated this data into the DES framework designed to provide users with the statistically enriched concepts in the topic-specific literature (in this case, about the amyloids and associated diseases), statistically “enriched pairs” of concepts or concepts that co-occur in text, as well as semantic similarity, for further exploration. We also provide an AD-related example to show how DES-Amyloidoses can assist researchers in the amyloid domain.

## 2. The DES-Amyloidoses exploration system

Efficient exploration of information related to amyloids is challenging as published information is substantial. For example, a PubMed Central query (31 December 2019) using "amyloid" retrieves more than 125,000 articles, of which 50% were published in the last five years. The challenge associated with exploring this voluminous amyloid-related information is further augmented when searching for links between different amyloids or between amyloids and other relevant biomedical concepts. However, the literature exploration process of complex biomedical topics of this nature has been made easy by developing several topic-specific KBs [64–75]. These KBs provide users with topic-specific enriched concepts and pre-computed statistically enriched associations between the enriched concepts. The text mining in these KBs are based on the titles and abstracts of publicly available PubMed records [32]. However, we can access significantly more information in full length articles [33]. Thus, the more recently developed topic-specific KBs use text mining based on both titles and abstracts (PubMed [60]) and full length articles (the subset of open-access articles from PubMed Central [37]). Unfortunately, to our knowledge, an amyloidoses-related KB of this type does not exist.

Thus, we developed DES-Amyloidoses as a topic-specific KB using an upgraded version of the Dragon Exploration System (DES), DES v3.0, on 17 February 2020. This version of DES allows users to explore scientific concepts through literature-derived topic-specific enriched concepts and pairs of concepts similar to the older versions, and it enables users to explore these concepts through semantic similarity. The underlying systems, and concept enrichment process used in the current version of DES has been described in [74].

### 2.1 The DES-Amyloidoses literature corpus

To create the DES-Amyloidoses literature corpus, on the 17 February 2020, we used the following query: *((Amyloid AND ("Serum Amyloid A" OR SAA OR "amyloid A" OR AA OR apoSAA OR ApoE OR "light chain amyloid" OR AL OR "amyloid enhancing factor" OR AEF OR "Serum amyloid P component" OR SAP OR glycosaminoglycans OR "Heparin sulfate proteoglycan" OR "islet amyloid polypeptide" OR IAPP OR "Imumunoglobin light chain" OR "Imumunoglobin heavy chain" OR AH OR Transyerthertin OR ATTR OR "β2-microglobulin" OR Aβ2M OR "Apolipoprotein AI" OR "AApo AI" OR AApoAII OR AApoAIII OR Gelsolin OR Agel OR Lysosyme OR Alys OR Fibrinogenα OR Afib OR "Cystatin C" OR ACys OR "ABriPP variants" OR ABri OR "α-Synuclein" OR AαSyn OR Tau OR ATau OR "Prion protein" OR APrP OR "Atrial natriuretic factor" OR AANF OR Prolactin OR "A Pro" OR Insulin OR AIns OR "Galectin 7" OR AGal7 OR Lactoferin OR ALac OR "Semenogelin 1" OR Asem1 OR Enfurvitide OR AEnf)) AND (human OR humans OR "homo sapiens"))*, to retrieve topic-specific articles from our local repository (MongoDB) of PubMed, and PubMed Central articles. This query retrieved 31,821 articles.

### 2.2 Dictionaries incorporated into DES-Amyloidoses

We imported 18 dictionaries from the pre-existing DES v2.0 vocabularies. To ensure completeness, we further compiled the topic-related dictionary, “Amyloids (Human and Mouse)” (see Table 1). The terms compiled in the 19 thematic dictionaries were normalized to a single internal identifier in the KB, where possible, to allow for more efficient mining of relevant terms in the text and enable linking terms to external sources. Additionally, term redundancies within the same dictionary are unified into one term. The text mining of most dictionary terms is generally straightforward. However, gene names are frequently interchangeably used with their protein product names/symbols in biomedical text. Thus, for example, in the "Human Genes and Proteins" dictionary, we combine EntrezGene [76] nomenclature (gene names/symbols) with UniProt [77] nomenclature (protein names/symbols). Also, concepts in all the dictionaries are normalized where possible, i.e., names/synonyms, and symbols referring to the same concept are retrieved by a single entity.

**Table 1:** DES-Amyloidoses dictionaries, terms per dictionary, and terms enriched in the literature corpus.

Dictionary	# Enriched concepts	# Enriched pairs of concepts	# Enriched pairs that contain amyloids	Status
**Chemicals/Compounds**				
Amyloids (Human and Mouse) [in-house compiled]	298	19,218	1,084	**newly compiled**
Chemical Entities of Biological Interest (ChEBI) [78]	6,996	233,926	1,769	pre-existing in DES
Lipids (Lipid Maps) [79, 80]	580	19,678	61	pre-existing in DES
Metabolites (MetaboLights) [81]	1,636	54,755	240	pre-existing in DES
Toxins (T3DB) [82]	1,046	47,615	314	pre-existing in DES
**Functional Annotation**				
Biological Process (GO) [83]	2,327	44,829	523	pre-existing in DES
Cellular Component (GO) [83]	645	17,404	201	pre-existing in DES
Molecular Function (GO) [83]	820	14,245	157	pre-existing in DES
Pathways (KEGG [84], Reactome [85], UniPathway [86], PANTHER [87])	723	18,773	144	pre-existing in DES
**Diseases**				
DOID Ontology (Bioportal) Human Disease Ontology [88]	1,517	33,970	312	pre-existing in DES
HP Ontology (Bioportal) Human Phenotype Ontology [89]	1,592	38,701	312	pre-existing in DES
SIDER (Drug Indications and Side Effects) [90]	1,354	31,828	276	pre-existing in DES
**Drugs**				
Drugs (DrugBank) [91]	1,887	76,994	531	pre-existing in DES
**Anatomies**				
Human Anatomy [in-house compiled]	1,325	76,315	1,001	pre-existing in DES
**Human**				
Human Genes & Proteins (EntrezGene) [92]	13,346	756,763	11,085	pre-existing in DES
Human Long Non-Coding RNAs [93]	99	2,232	20	pre-existing in DES
Human microRNAs [93, 94]	741	26,002	61	pre-existing in DES
Human Transcription Factors [95]	1,010	63,353	647	pre-existing in DES
Mutations (tmVar) [96]	5,144	40,204	480	pre-existing in DES

Initial indexing is performed, that is, concepts in these dictionaries are mined in the prepared literature corpus and color-coded to reflect the dictionary from which it was retrieved. In this manner, we can identify 1/ promiscuous terms through their high frequencies due to their use as ordinary English words, and 2/ terms in newly imported dictionaries not found in the prepared literature corpus, which we exclude as part of the dictionary cleaning process. Then, re-indexing is performed based on clean dictionary data.

Table 1 lists the dictionaries used and provide, 1/ the number of enriched concepts in the literature corpus per dictionary, 2/ the number of enriched concept pairs in the literature corpus per dictionary, and 3/ the number of enriched pairs that include an amyloid per dictionary. Out of all concepts in the 19 dictionaries, 43,086 were found to be statistically enriched. Based on the statistically enriched terms in the corpus, the system identified 796,409 enriched pairs of concepts in the literature corpus. Embedding the network of concept pairs enabled semantic similarity computation between the KB concepts.

#### 2.2.1 Enriched concepts

The frequency at which a concept appears in the full literature set is expected to be similar to its frequency in that literature’s random subset. Thus, in DES, a concept is defined as enriched when overrepresented in the topic-specific corpus, in this case, the DES-Amyloidoses corpus, compared to the complete set of PubMed and PubMed Central articles in DES MongoDB database. We calculated the false discovery rate (FDR) <0.05 (*P-value*) based on the Benjamini–Hochberg procedure to correct for multiplicity testing. Concepts are quantified to be enriched when it has an FDR/*P-value* < 0.05 in the DES-Amyloidoses corpus compared to the complete article set. In this manner, the KB provides the user with the most topic-relevant concepts.

#### 2.2.2 Enriched pairs

The enriched or topic-relevant concepts co-occur in literature with several other concepts. We classified concepts as co-occurring if they were mentioned within a 200-character distance in text. This co-occurrence of concepts may also be enriched; for example, the enriched concept may co-occur with the other concept 90% of the time. Thus, DES-Amyloidoses also provides users with the pairs of enriched concepts based on co-occurrence (or association) compared to the enriched concept’s occurrence (the second concept in the pair may or may not be enriched). The concepts’ co-occurrence signifies a potential association, but concepts in the enriched pair might not be directly associated. Nonetheless, enriched concept pairs increase the probability of an association between the two concepts existing.

#### 2.2.3 Semantic similarity

Here, we used semantic similarity as a metric that establishes how close in meaning or relatedness two concepts are, based on their distribution within a text corpus. The semantic similarity relatedness can be in the form of hypernymy/hyponymy, antonymy, or synonymy. For example, liquid and water are semantically similar even though they are different concepts because water is a hyponym of liquid, and hence are more likely to be co-mentioned in the same context. We acquired the semantic similarity by first training a skip-gram Word2Vec model on the DES-Amyloidoses corpus, then calculating the cosine distance between concept embeddings, representing semantic similarity in DES. Therefore, semantic similarity represents a concept co-occurrence in DES, which might not be direct.

## 3. DES-Amyloidoses utilities and case study

DES-Amyloidoses provide users with a list of topic-specific enriched concepts and lists of concepts frequently mentioned in the same text as the enriched concept based on the amyloid-related literature, as these concepts may be directly or indirectly associated with the enriched concept. Users can explore these enriched concepts via multiple links built into the KB, including “Enriched Concepts", “Enriched Pairs”, and “Semantic Similarity” (described in detail by [70, 74]).

Briefly, the “Enriched Concepts” link allows users to familiarize themselves with and explore the concepts enriched in the amyloid-related literature, such as APP, amyloid-beta, MAPT, cerebral, etc. The “Enriched Pairs” link allows the users to focus on a specific enriched concept of their interest, and explore other concepts (not enriched in the amyloid-related literature) that may be associated with the enriched concept, for example, NLRP3 and aortic sinus; NLRP3 and saturated fatty acid anion; NLRP3 and cytochalasin; NLRP3 and LRRFIP1, and so forth. On the other hand, the “Semantic Similarity” link allows the users to explore concept co-occurrence that is not necessarily directly linked, as in the “Enriched Pairs” case; checking such co-occurring concepts for inferred biological association can be used to shortlist potential novel hypotheses.

For each exploration, users can view enriched concepts in pre-compiled theme-based dictionaries and restrict concepts based on a specific term/concept, for example, using the text box to search for the enriched concepts that contain the term Amyloid, retrieves concepts such as “Conjunctival amyloidosis”, “paramyloidosis”, “amyloid precursor protein metabolic process”, and so forth. Concepts of interest can also be sorted using ranking options, including false discovery rate (FDR), density, KB frequency (KB_FDR), and background frequency (BKB_FDR) (see “Column visibility”), and results can be exported in excel or csv format via the “Export” link. Also, each concept is linked to a hover box from which users can generate a “Network” or retrieve “Term Co-occurrences”; generated networks can be saved in the json.txt format using the “Export Network” link.

### 3.1 Case study 1: Illustrating the usefulness of DES-Amyloidoses as a research support system: Progression of an Amyloid “network” in the pathogenesis of AD

Here we demonstrate the efficacy of DES-Amyloidoses in exploring inflammasome-amyloid associations, as amyloids have been demonstrated to activate the inflammasome to process Interleukin 1 beta (IL-1β) [97]. For example, activation is induced in AD via Aβ [98], in Diabetes type II (T2D) via IAPP [99], in PD via α-synuclein [100] and in amyotrophic lateral sclerosis (ALS) through SOD1 [101].

**Fig 1:**
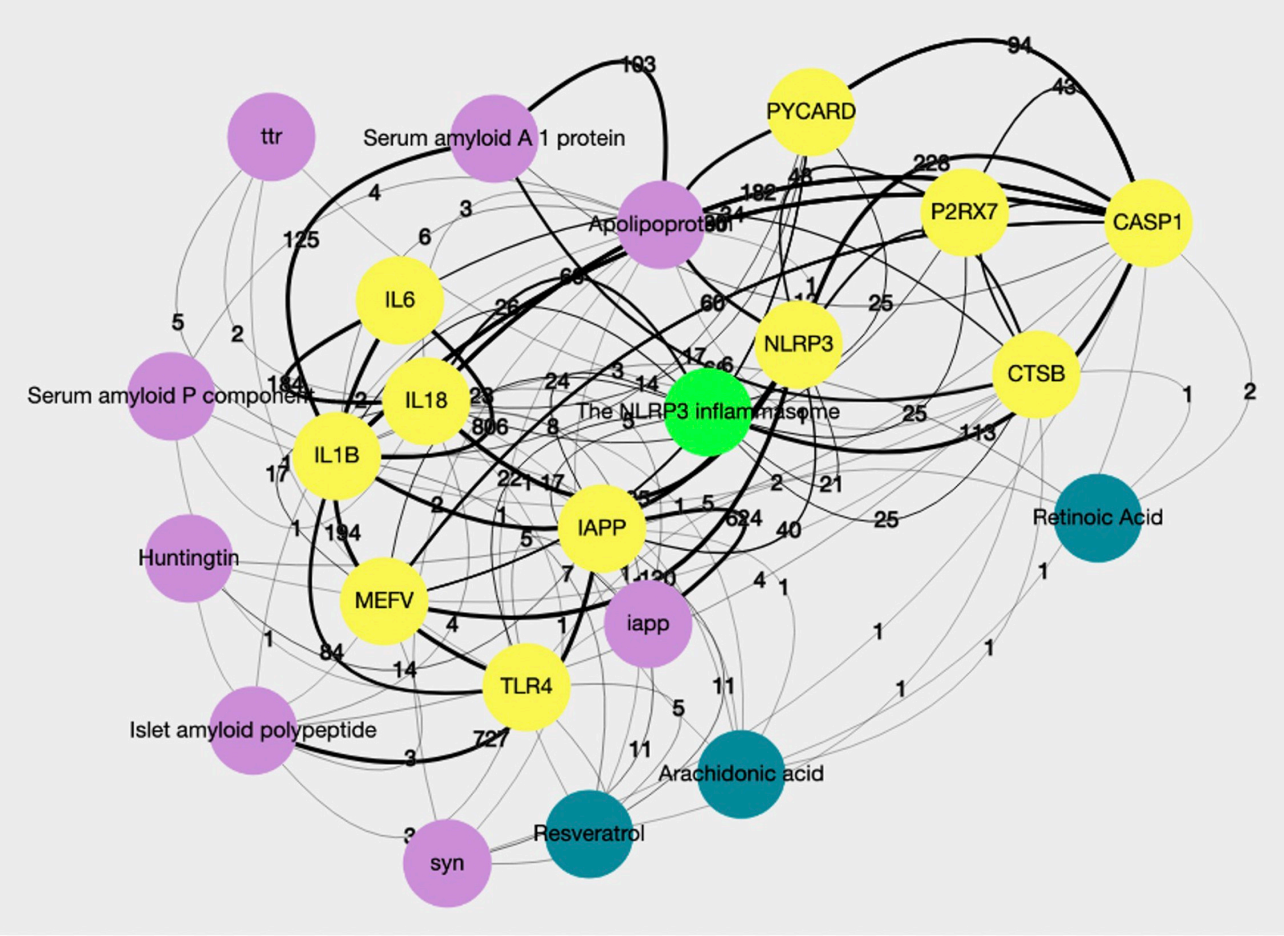
A depiction of the inflammasome-amyloid “network” involved in Alzheimer’s disease’s pathogenesis.

We started this process by checking if the inflammasome pathway is an enriched concept in the amyloid literature. This was done by clicking the DES-Amyloidoses “Enriched Pairs” option (Step 1), which opens a page that lists associated terms from all dictionaries in two columns. In the first column, where users can specify the first dictionary (or concept A), we filtered by selecting the “Human Genes and Proteins (EntrezGene)" dictionary from the drop-down menu. Similarly, for the second dictionary (or concept B), we selected the “Pathways” dictionary from the drop-down menu. The “inflammasome” pathway is listed multiple times, and even the “The NLRP3 inflammasome” pathway is listed as one of the most significant pathways. Because Halle *et al*. [98] demonstrated that activation of the NALP3 inflammasome is an essential process in AD-related inflammation and tissue damage, we proceed by accessing the right-click menu (or hovering over) for the “The NLRP3 inflammasome” concept to generate a network (Step 2). On the network page, we selected "Amyloids", "Human Genes and Proteins", and "Lipids" in the “Choose Dictionaries” menu, then selected the ‘The NLRP3 inflammasome’ node and used the right-click menu to ‘Expand’ the network with nodes from the selected dictionaries (Step 3). Using the same dictionaries, we performed a second round of network expansion on all nodes obtained in Step 3. We removed all nodes with two or fewer links (Fig 1, Step 4).

The final network comprises two sub-networks; one centered on the NLPR3 inflammasome node, while the other is centered on the amyloid, IAPP. The amyloid network, in this case, IAPP, works in concert with the activation of the inflammasome through NLPR3. Moreover, it also depicts an array of inflammation-related genes/proteins, and a direct association between NLPR3-CASP1(inflammation-associated protein) and TRIM20 (MEFV) associated with innate regulation immunity suggests crosstalk between amyloids, innate immunity, and inflammation. Specifically, MEFV inhibits the NLPR3-CASP1 inflammasome pathway by directly binding to inflammasome components, including NLRP1, NLRP3, and CASP1. Also, it recruits autophagic machinery to execute degradation [102]. In this manner, autophagy controls the hub signaling machinery [102]. A similar direct association is depicted between NLPR3-CASP1 (inflammation-associated proteins) and Cathepsin B (CTSB). CTSB plays a crucial role in several physiological processes, one essential being driving degradation within the lysosome [103]. CTSB reduces the expression levels of lysosomal and autophagy-related proteins, thereby reducing the number of lysosomes and autophagosomes in the cell. It has further been demonstrated that CTSB is released from the lysosome with lysosomal damage, causing autophagy-lysosomal dysfunction and the activation of the NLRP3-CASP1 inflammasome pathway [98, 104, 105]. Thus, both IAPP and Aβ induce NLPR3-CASP1 activation through a mechanism involving the released CTSB [99].

The precise mechanism of NLPR3-CASP1 activation is still debated, however, considering that 1/ CTSB inhibition prevents Aβ-induced NLPR3-CASP1 activation, which reduces amyloid plaque load and improves memory in the AD brain of mouse models [106], and 2/ CTSB has been associated with several amyloids, this network provides users with a bird’s-eye view of amyloid-related literature. It suggests CTSB should be considered a potential therapeutic approach for treating AD wherein the inflammasome is targeted.

### 3.2 Case study 2: DES-Amyloidoses unveils the microRNA, possibly regulating the Amyloid “network” in the pathogenesis of AD

**Fig 2:**
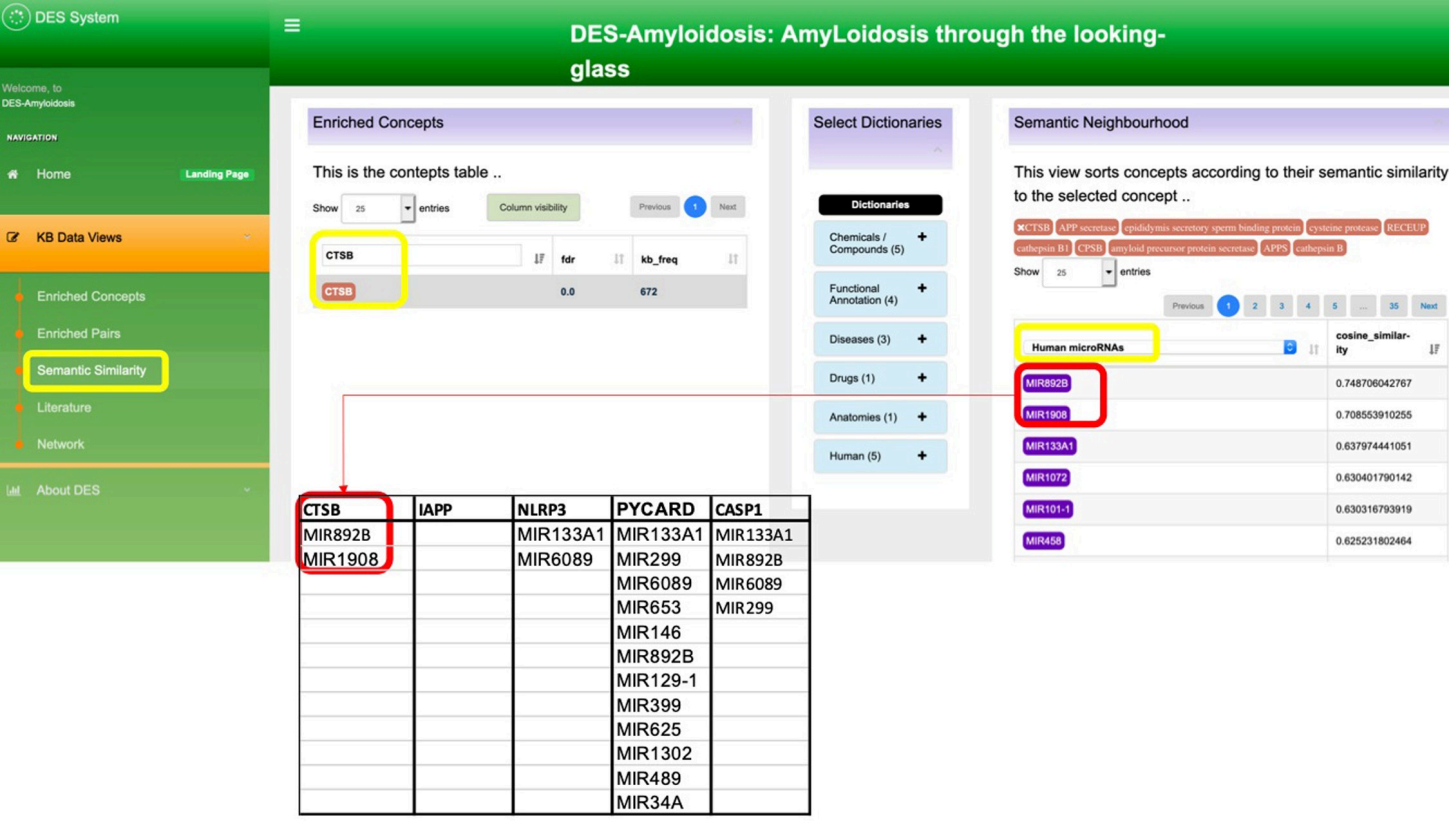
An illustration of how DES-Amyloidoses can be used to identify relationships between the concepts based on semantic similarity. The yellow square indicates the changes that were implemented, and the tabulation shows the microRNAs that were shortlisted for this process.

To identify the microRNAs possibly regulating the inflammasome-amyloid associations in AD, we explored the microRNAs semantically linked to the essential genes ("IAAP," "CTSB," "NLRP3", "PYCARD," and "CASP1") identified in the inflammasome-amyloid associations in AD (see case study 1). This was done by clicking the DES-Amyloidoses “Semantic Similarity” option, which opens a page with two columns. In the first column, we inserted the name of the gene of interest (or concept A), but for the second column, we selected the “Human microRNAs” dictionary from the drop-down menu (or concept B) (see Fig 2). Then, we repeated this process for all genes of interest and tabulated all microRNAs significantly linked to these genes (provided in Fig 2).

**Fig 3:**
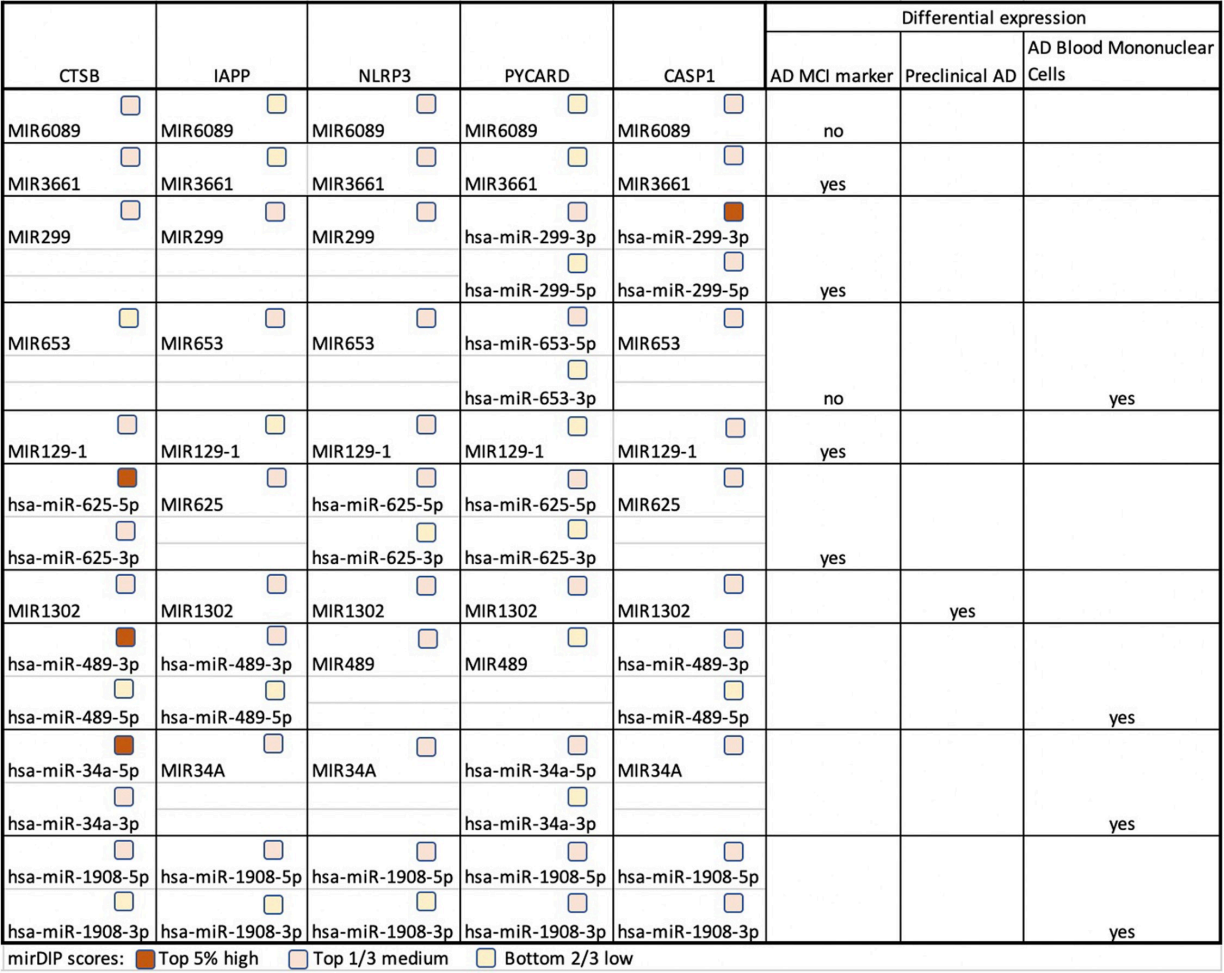
The microRNAs predicted to target the essential genes with the mirDIP scores indicated. AD MCI marker [110]; Preclinical AD [107]; AD Blood Mononuclear Cells [108, 109].

We identified 13 unique microRNA with cosine similarity above 0.7. However, we found no literature connecting these microRNA to the amyloid “network” despite this indirect association depicted by DES-Amyloidoses. Consequently, we used the microRNA Data Integration Portal (mirDIP) to search if these microRNAs are predicted to target our set of genes. As a result, we found 9 of the 13 unique microRNA (hsa-miR-6089, hsa-miR-3661, hsa-miR-299, hsa-miR-653, hsa-miR-129-1, hsa-miR-625, hsa-miR-1302, hsa-miR-489, hsa-miR-34a, and hsa-miR-1908) predicted to target all five genes, and a literature search showed all these microRNAs have differential expression linked to AD [107–110] (see Fig 3). Furthermore, one of the microRNAs, hsa-miR-1908, was experimentally validated to inhibit ApoE expression, which suggested that miR-1908 inhibits Aβ clearance by repressing ApoE expression [111].

## 4. Discussion

The idea that the amyloids present a system is not new [112–114]. The reason is that they have networks that suggest interrelations with other biological networks and environmental stressors that can induce metabolic changes that may impair homeostatic defenses during the lifetime of humans. Furthermore, changes in the interrelations with these other networks may cause differences in patterns, heterogeneity, age of onset, disease progression, and divergent patient phenotypes. Some phenotypes have emerged due to different Aβ conformations [115] and the seeding capabilities of the amyloids [38, 116], which adds to this complexity. For example, IAPP was identified in human cerebral Aβ deposits, and Aβ fibrils were found to seed IAPP in vivo as efficiently as hproIAPP [116], which offers a possible molecular link as to why epidemiological studies suggest patients with type 2 diabetes have an almost twofold increased risk of developing AD [117].

Specific amyloid converting endotrophic triggers have not yet been pinpointed, despite genetic mutations linked to some disorders’ etiology. The reason is that sensitivity towards environmental pathogens (e.g., pesticides), reactive oxygen species, or metals characterizes amyloid aggregation, which partially explains the idiopathic amyloid disorders, is more frequent than familial cases. Fig 1 suggests that IAPP being more prone to Aβ Amyloidosis than AA amyloidosis may be a consequence of the divergence in its interaction with the NLRP3-inflammasome that can sense and respond to dysfunction triggered by environmental stressors [114].

Clearance of amyloids by phagocytosis is a needed physiological process, but it can adversely perturb cellular homeostasis. Specifically, phagocytosis of amyloid peptides, like Aβ and IAPP, still may lead to the activation of the innate immunity activator, NLPR3 [118]. A recent paper by Cai and colleagues [119] showed that pattern recognition receptors and prion could replace NLPR3 and ASC, respectively, in inflammasome signaling. This may indicate that amyloids have a more intrinsic role in inflammatory processes than previously realized, and they may be working with other signaling proteins to shift perturbed homeostatic mechanisms to typical values, which suggests a protective role. On the other hand, these amyloids may independently aggravate inflammation in neurodegeneration disorders such as AD by activating caspase 1, which then cleaves pro-IL-1β and pro-IL-18 into their mature, secreted forms resulting in neuronal cell death [120]. A multitarget approach may be a promising therapy strategy; as the case studies indicate, targeting Cathepsin B in concert with an inflammasome-amyloid network associated microRNA/s may reset the mechanism altered in AD.

## 5. Concluding remarks

The notion that amyloids comprise a “Network” that can be defined as a system is an essential jump in understanding protein folding diseases. By defining the network/system in which a disease is presented through an integrative perspective from the genotype to the phenotype, it shall be possible to discern important “hub” proteins and pathways, as shown through the “inflammasome-amyloid hub”. With DES-Amyloidoses we presented an “amyloid system” and its interacting network based on the literature and data mining approaches compiled into a KB. This system enables a novel way to interrogate information about amyloids and associated diseases. We hope the two case studies shared demonstrate how users may find DES-Amyloidoses to be a valuable tool for supporting amyloidoses-related research questions. Furthermore, we intend to update the KB biannually to ensure the KB contents remain current.
